# Iodine Biogeochemical Cycle and Microbial Bioremediation of Radioactive Iodine-129

**DOI:** 10.4014/jmb.2508.08018

**Published:** 2025-09-26

**Authors:** Hwa-Hyung Lee

**Affiliations:** Radiation Safety Center, Kyungpook National University, Daegu 41566, Republic of Korea

**Keywords:** Iodine biogeochemical cycle, radioactive Iodine-129, microbial bioremediation, bioreduction, biosorption and bioaccumulation, biomineralization

## Abstract

Iodine is an essential biophilic element that plays pivotal roles in both environmental systems and human physiology, particularly as a key constituent of thyroid hormones and a regulator of atmospheric ozone. In contrast, its radioactive isotope, iodine-129 (I-129), predominantly generated through anthropogenic nuclear activities, represents a persistent environmental and public health concern. With an exceptionally long half-life of approximately 15.7 million years and high environmental mobility, especially in groundwater, combined with a strong tendency to bioaccumulate in the human thyroid, I-129 poses a disproportionate and long-term radiological hazard in contaminated sites. The biogeochemical cycling of iodine involves intricate interconversions among multiple oxidation states and phases across the lithosphere, hydrosphere, atmosphere, and biosphere. Microorganisms are central to these processes, mediating oxidation, reduction, methylation, accumulation, and sorption. While microbial methylation can increase I-129 mobility via the production of volatile methyl iodide, other microbial pathways, notably biosorption and binding to organic matter, provide promising mechanisms for immobilization and natural attenuation. Microbial bioremediation offers a sustainable and cost-effective alternative or complement to conventional physicochemical methods for managing radioactive contaminants. Strategies such as bioreduction, biosorption, bioaccumulation, and biomineralization exploit the metabolic versatility of microorganisms to alter radionuclide speciation, solubility, and mobility. However, practical application to I-129 remains challenging due to its extreme persistence, environmental variability, and uncertainties in predicting its long-term geochemical fate. Effective management of I-129 contamination will require an integrated, multidisciplinary approach that combines advanced microbial ecology insights, optimized biotechnological processes, and long-term monitoring frameworks.

## Introduction

Iodine is widely recognized as a biophilic element, signifying its strong affinity for biological systems and its extensive involvement in life processes [1]. This element is fundamental to the metabolism of numerous living organisms across the three major domains of life [1, 2]. Its presence in high amounts in the oceans, where life is believed to have originated, further supports its ancient role in the biology of emerging organisms [1-3]. The chemical speciation of iodine is a primary determinant of its availability to living organisms and its subsequent biological activities [1-4].

Beyond its fundamental biological role as an essential micronutrient for human health, iodine is indispensable for the synthesis of thyroid hormones, including triiodothyronine (T3) and thyroxine (T4), which are crucial regulators of metabolism and vital for cognitive development [5]. The primary sources of iodine for human consumption are food, particularly vegetables grown in iodine-rich soils, and marine products such as various seaweeds (like wakame, nori, or mekabu), which are rich sources of this element [5].

In addition to its biological significance, iodine has a profound influence on atmospheric chemistry, playing a crucial role in Earth's atmospheric processes. Marine iodine, specifically the reduced iodide ion (I^-^), plays a key role in controlling atmospheric ozone levels [6]. The rapid reaction between sea-surface iodide and ozone is considered the single largest natural source of gaseous iodine to the atmosphere [7]. Once in the atmosphere, reactive iodine species contribute significantly to ozone destruction. For instance, they are responsible for approximately 15% of tropospheric ozone loss and contribute up to 4.2% of stratospheric ozone depletion over the Antarctic "ozone hole" [6]. This demonstrates a powerful causal link between local biogeochemical processes, such as the iodine cycle in the marine environment, and the broader global atmosphere. The evolving marine iodine cycle has fundamentally shaped the stability and abundance of the ozone layer for approximately two billion years following the initial oxygenation of the atmosphere [6]. This highlights the necessity for a holistic understanding of elemental cycles, as perturbations in one compartment, such as ocean chemistry influenced by climate change, can have cascading effects on global atmospheric health and climate, demanding integrated environmental management strategies.

This complex biogeochemical cycle of iodine is governed by the continuous interaction of biotic and abiotic transformations across the terrestrial, hydrosphere, atmosphere, and biosphere, with microbial activity playing a central role in redox reactions, methylation, accumulation, adsorption, and often motility-enhancing roles [8].

This inherent biophilic nature, while essential for the stable isotope of iodine, presents a significant challenge when considering its radioactive counterpart, iodine-129 (I-129, ^129^I). Iodine-129 is a long-lived radioisotope of iodine with a half-life of approximately 15.7 million years [9]. Its exceptionally long half-life makes it a radioactive nuclide of particular importance in the long-term management of spent nuclear fuel and radioactive waste [9]. The very mechanisms that facilitate the uptake of stable iodine, such as active transport systems in the thyroid gland, also readily incorporate I-129 into biological systems [5]. This leads to a pronounced bioaccumulation of I-129 in the human thyroid gland, where approximately 90% of the body's iodine concentrates [5]. Consequently, the natural biological affinity for iodine transforms into a critical vulnerability, as the incorporated radioiodine can act as a potential carcinogen, posing a substantial risk of cancer [5]. Therefore, the characteristic that makes stable iodine vital for human health simultaneously renders its radioactive isotope a particularly challenging contaminant, necessitating remediation strategies that not only transform its chemical species but also effectively reduce its bioavailability and uptake into the food chain.

Microorganisms play a central role in the iodine biogeochemical cycle, actively mediating key transformation processes, including oxidation, reduction, methylation, accumulation, and sorption [8]. While microbial methylation can unfortunately enhance the mobility of I-129 by producing volatile methyl iodide, other microbial activities, particularly sorption and binding to organic matter, offer promising pathways for its immobilization and natural attenuation [8]. Microbial bioremediation offers an environmentally friendly and cost-effective alternative or complement to conventional physicochemical methods for managing radioactive contaminants such as radioactive I-129.

In this review, the biogeochemical cycle of iodine is intensively discussed, including the interplay of its biotic and abiotic cycling characteristics. In addition, the environmental impact of radioactive iodine-129 was discussed, as well as the recent advances in the microbial bioremediation of iodine-129. Lastly, the challenges and future directions of microbial bioremediation for Iodine-129 were also discussed in relation to long-term stewardship strategies that manage and mitigate risk over geological time scales in this review.

## The Biogeochemical Cycle of Iodine

**Fig. 1:**
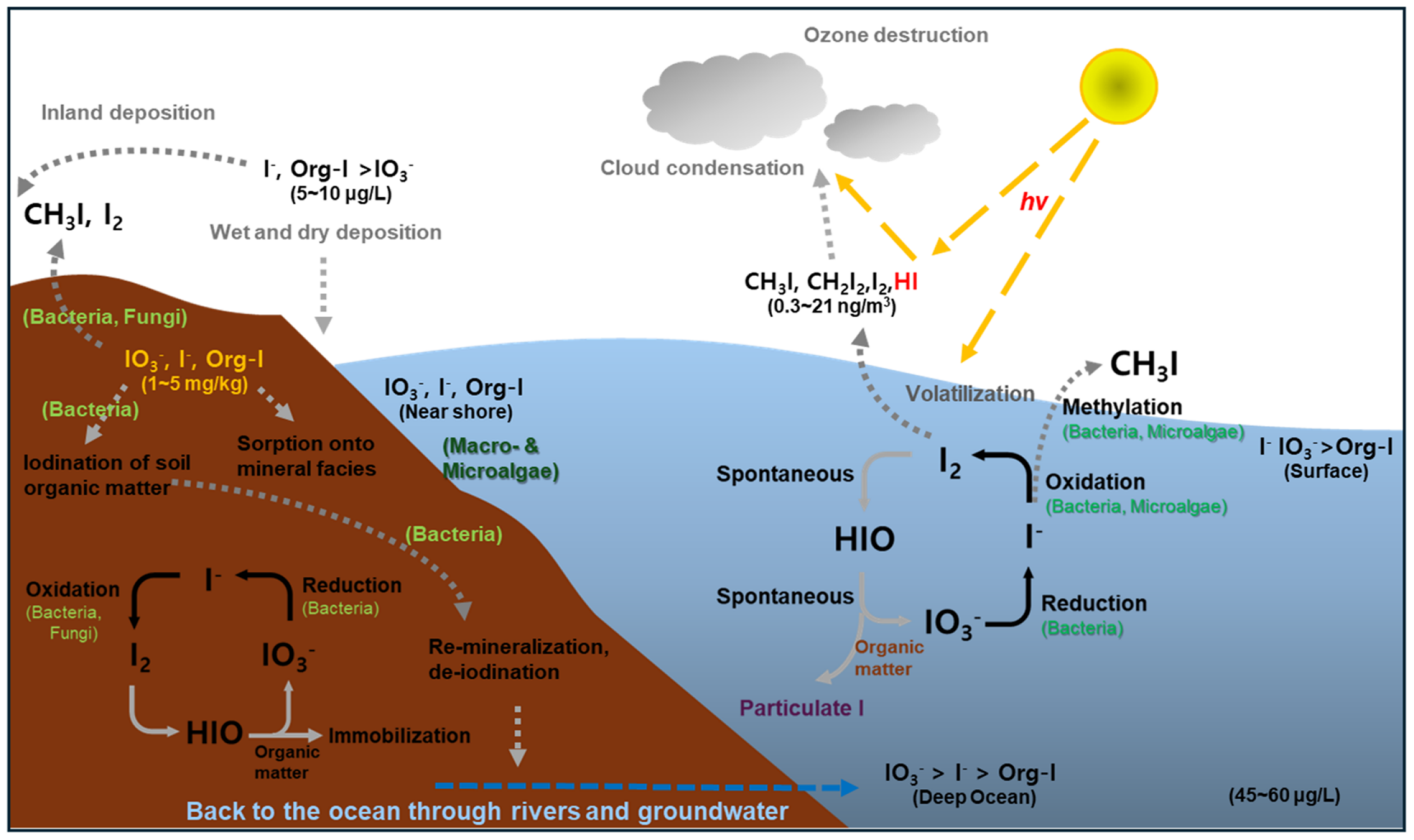
Global iodine biogeochemical cycle. Marine systems serve as the primary reservoir for iodine, which exists primarily as iodide (I^−^), iodate IO_3_ −, and organic iodine (org-I). Biological and photochemical processes act to volatilize iodine, releasing I_2_, HI, and org-I (such as methyl iodide) species into the atmosphere, where additional photochemistry transforms iodine into reactive species and/or particles capable of ozone destruction and acting as nucleation points for cloud condensation [10]. Dry and wet deposition of oceanic iodine serves as the primary source of iodine for the surface terrosphere [10]. Re-volatilization of iodine from soils and freshwaters near the coast enables movement of iodine further inland [10]. Soils serve as a critical iodine sink, primarily in the form of relatively immobile org-I. Micro- and macroalgae, fungi, and bacteria play a central role in the iodine redox cycle by oxidizing I^−^ to I_2_ and IO_3_^−^, formation of org-I, dehalogenation of org-I, and reduction of IO_3_^−^ to I^−^ [10]. Reactive intermediates formed during IO_3_^−^ reduction, and particularly I^−^ oxidation, can form covalent bonds with organic matter, leading to the formation of org-I, which readily binds to particles in the ocean, atmosphere, and soil environments [10].

The biogeochemical cycle of iodine is a dynamic and intricate system characterized by the exchange and transformation of iodine across the Earth's major reservoirs: the lithosphere, hydrosphere, atmosphere, and biosphere (Fig. 1). The mobility of iodine in the environment is intricately controlled by its chemical speciation, which is itself governed by a complex interplay of redox reactions, complexation, sorption, precipitation, and significant microbial activities [10]. Over the past 15 years, renewed interest in the environmental fate of radioiodine has significantly advanced the understanding of iodine biogeochemistry, particularly the role of these various processes [10].

### Major Reservoirs and Forms of Iodine in the Environment

Iodine exists in multiple oxidation states, ranging from -1 to +7, and is found in various chemical forms, including inorganic salts (iodides and iodates), molecular iodine (I_2_), hypoiodous acid (HIO), and a diverse array of organic iodine compounds [2]. Specific forms of iodine determine their behavior and mobility within different environmental compartments.

**Lithosphere:** The Earth's crust is a significant reservoir of iodine, with the richest inorganic sources found in oceanic sediments (68.2%) and continental sedimentary rocks (27.7%), as shown in Table 1 [2]. Igneous and metamorphic rocks contribute smaller amounts (2.7%), as does the mafic oceanic crust (0.68%), as shown in Table 1 [2]. Soil, in contrast, naturally contains very little iodine; its deposition primarily occurs through volatilization from ocean water, a process aided by ultraviolet radiation [3, 5]. Within soils, a substantial portion of iodine becomes bound to organic and inorganic matter, a process often influenced by microbial activity [11].

**Hydrosphere:** Oceans represent the world's largest repository of iodine [3, 5]. The total iodine concentration in the modern ocean is generally conservative, typically ranging from 500 nM to all depths [6, 12]. In seawater, iodate (IO_3_^-^) and iodide (I^-^) are the dominant dissolved anionic forms, with iodate comprising over 80% in the modern ocean, as shown in Table 1 [6, 12]. Under low marine oxygen conditions, iodate undergoes a near-complete reduction to iodide [6, 12]. Iodide also accumulates at lower concentrations in euphotic waters due to primary production by phytoplankton [6, 12]. Within seawater, iodine species are dynamically cycled through processes such as the subduction of oceanic crust and the decomposition of marine organisms [2].

**Atmosphere:** Iodine emissions to the atmosphere originate from several sources, including sea spray aerosolization, volcanic gases, human activities, and, notably, from biological conversion to volatile methyl forms, predominantly methyl iodide (CH_3_I) [2]. Once in the atmosphere, emitted I_2_ gas can be photolyzed by both UV and visible wavelengths, producing atomic iodine, which then participates in catalytic cycles that scavenge ozone [6]. Atmospheric iodine eventually returns to the lithosphere through wet and dry deposition, completing a part of its global cycle [11].

**Biosphere:** A wide array of living organisms actively participate in the cycling of iodine. Algae, plants, corals, sponges, anemones, lobworms, shellfish, arthropods, and bacteria all accumulate and cycle both organic and inorganic iodine species [2]. Terrestrial plants absorb iodine from the soil through their roots and return it to the environment upon decomposition [11, 12].

The prevailing redox potential (Eh) of an environment is a critical determinant of iodine's chemical form and, consequently, its behavior, transport, and bioavailability. This is evident from the fact that iodide (I^-^) is identified as having the highest mobility in terrestrial environments [13], while iodate (IO_3_^-^) is generally less mobile and the dominant form in oxic oceans [6]. The observation that low oxygen conditions in marine environments lead to the reduction of iodate to iodide directly illustrates this dependence [6]. This fundamental relationship means that understanding and manipulating these redox transformations are central to managing both stable iodine distribution and radioactive iodine contamination.

### Iodine Transformation

The mobility of iodine in the environment is intricately controlled by its speciation, which itself is governed by a dynamic interplay of redox reactions, complexation, sorption, precipitation, and significant microbial activities [10-12]. Over the past 15 years, renewed interest in the environmental fate of radioiodine has significantly advanced our understanding of iodine biogeochemistry [10-12].

**Oxidation and reduction mechanisms.**

Microorganisms play a central role in the redox transformations of iodine, significantly influencing its mobility and bioavailability [11].

**Fig. 2:**
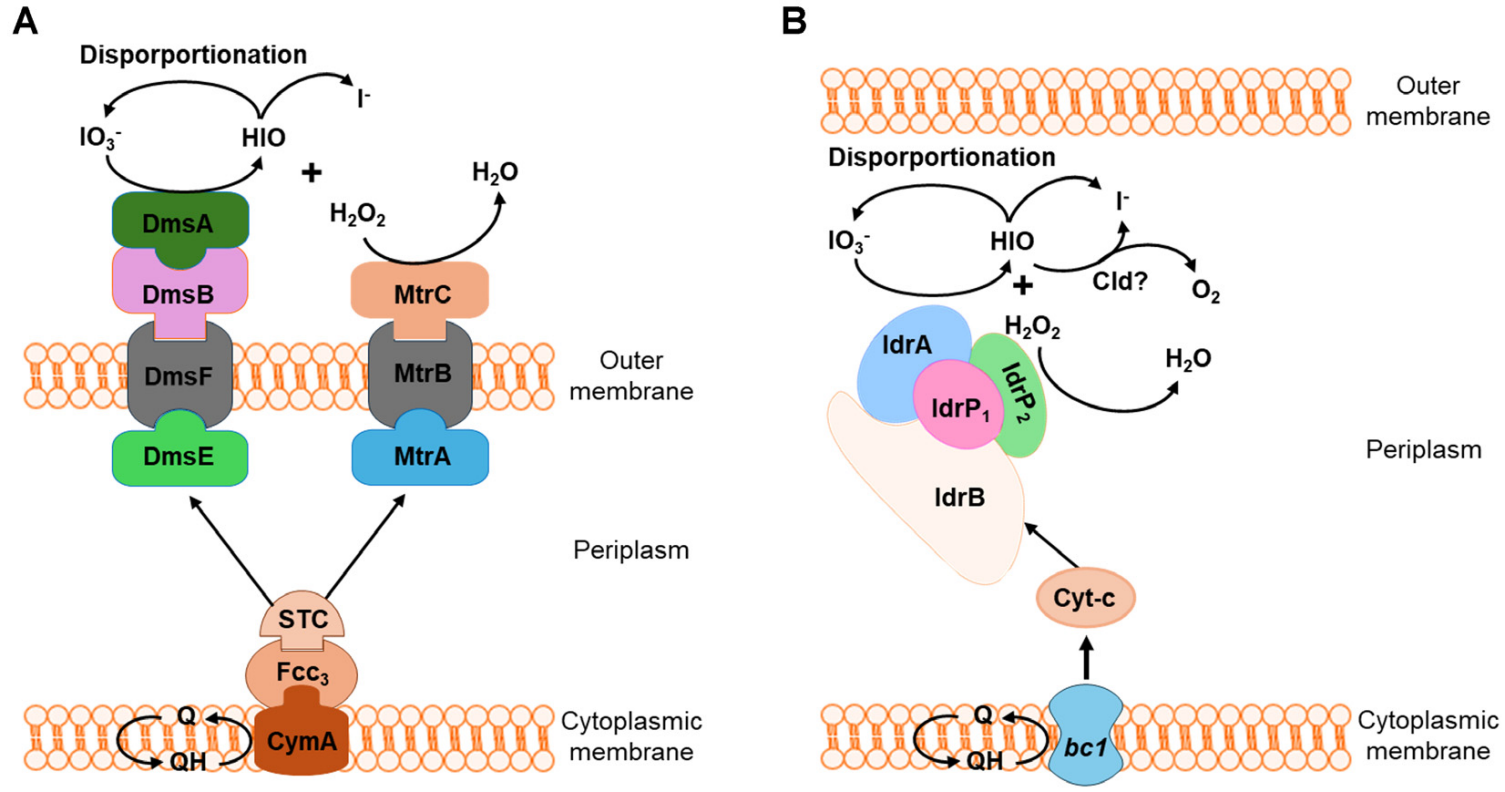
Molecular mechanisms for the bacterial reduction of iodate. (**A**) Extracellular reduction by DmsEFAB and MtrCAB in sulfur and iron-reducing bacterium *Shewanella oneidensis* [14-17] and (**B**) Periplasmic reduction by IdrABP1P2 in iodate-reducing bacterium *Pseudomonas* sp. SCT [18].

**Microbial reduction:** Dissimilatory IO_3_^-^-reducing bacteria actively reduce iodate (IO_3_^-^) to iodide (I^-^) under anoxic conditions [10]. Fig. 2 shows the molecular mechanisms for the bacterial reduction of iodate. This process is mediated by specific iodate reductases, including periplasmic iodate reductase IdrABP1P2, extracellular DMSO reductase DmsEFAB, and metal reductase MtrCAB [10, 14-17]. These enzymes initially reduce IO_3_^-^ to hypoiodous acid (HIO) and hydrogen peroxide (H_2_O_2_); HIO is then proposed to disproportionate abiotically into I^-^ and IO_3_^-^ at a 2:1 ratio [10, 14-17]. Furthermore, H_2_O_2_ is reduced to H_2_O by IdrP1P2 and MtrCAB as a detoxification mechanism [10, 14-18]. Dissimilatory Fe(III)- and sulfate-reducing bacteria also contribute to IO_3_^-^ reduction, either directly via their reductases or indirectly through the abiotic reduction of Fe(II) and sulfide, their metabolic byproducts [14-17]. This microbial reduction of iodate to iodide has been observed in various environments, including marine microalgae, bacteria, and during the early stages of metal reduction in sediment microcosms [10]. Specific examples of bacteria capable of this include *Pseudomonas* sp. SCT, *Shewanella oneidensis*, *Desulfovibrio desulfuricans*, and *Shewanella putrefaciens*, which highlight their role in mediating both direct enzymatic and indirect abiotic reduction of iodate in anaerobic environments [14-17, 19]. The reduction of iodate to iodide is particularly significant because iodide is generally more mobile than iodate, meaning microbial reduction can directly increase iodine's environmental dispersion.

**Fig. 3:**
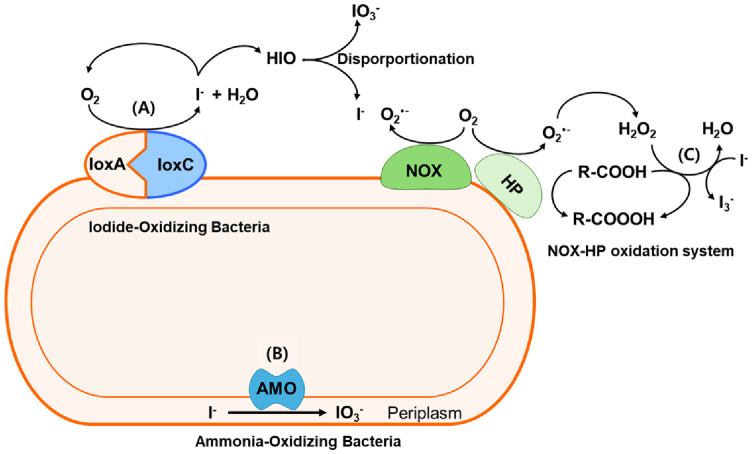
Bacterial oxidation of iodide. (**A**) Extracellular multicopper iodide oxidase of iodide-oxidizing bacteria; (**B**) Periplasmic ammonia monooxygenase of ammonia-oxidizing bacteria; (**C**) NOX-HP oxidation system generating extracellular reactive oxygen species. Abbreviations: Amo: ammonia monooxygenase; HP, heme peroxidase; Iox: iodide oxidase; NOX, NADPH oxidase.

**Microbial oxidation:** Conversely, iodide (I^-^)-oxidizing bacteria facilitate the oxidation of I^-^ to molecular iodine (I_2_) directly under oxic conditions, primarily through their extracellular multicopper iodide oxidases (IoxAC) [10, 20-22] from iodide (I^-^)-oxidizing bacteria, such as *Iodidimonas* sp. Q-1 and *Roseovarius* sp. strain A-2 producing a variety of organic iodine compounds during I^-^ oxidation [10, 20-22]. Ammonia-oxidizing bacteria, such as *Nitrosomonas* sp. Nm51 and *Nitrosococcus oceani* Nc10 are also capable of oxidizing I^-^ to IO_3_^-^ directly under oxic conditions, likely utilizing their intracellular ammonia-oxidizing enzymes [10, 22]. Additionally, many bacteria produce extracellular reactive oxygen species through NADPH oxidase (NOX), which can oxidize I^-^ to triiodide (I_3_^-^) by Heme peroxidase (HP) [10, 22]. Fig. 3 displays an overview of bacterial iodide oxidation pathways, which are the extracellular multicopper iodide oxidase of iodide-oxidizing bacteria, the periplasmic ammonia monooxygenase of ammonia-oxidizing bacteria, and the NOX-HP oxidation system.

While the abiotic oxidation of I^-^ to I_2_ or HIO occurs very slowly due to iodide's stability in typical soil pH and Eh conditions, multiple studies have demonstrated that microorganisms and/or their enzymes significantly improve the kinetics of this reaction [10, 22]. Extracellular oxidases, predominantly of bacterial origin, are identified as key catalysts for soil iodination in aerobic, surface soils of deciduous and coniferous forests [10, 20-22]. Microorganisms are not merely passive agents but actively improve the kinetics of these redox reactions. This suggests that microbial activity acts as a critical switch, determining whether iodine, including the radioactive isotope I-129, is transported and dispersed within environmental compartments or fixed and retained. This dynamic control is central to understanding and managing iodine's ecological fate [10, 22].

**Fig. 4:**
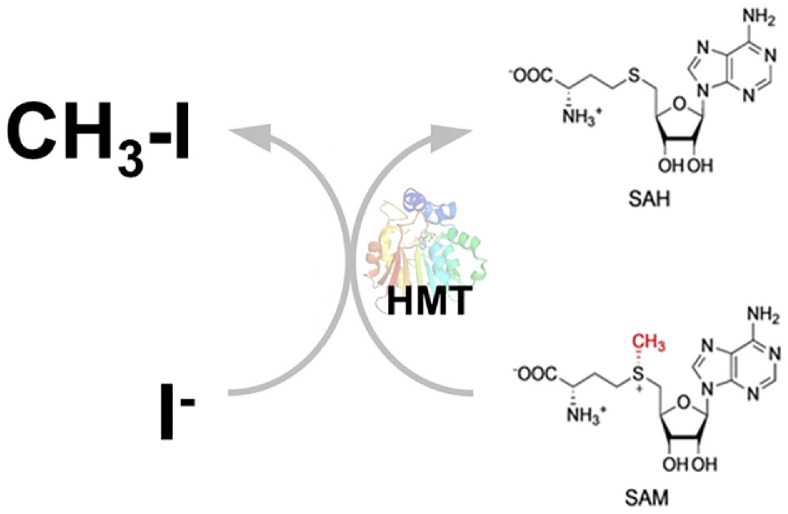
Biosynthesis of methyl iodide from inorganic iodide by bacterial SAM-dependent halide methyltransferase (HMT). Bacterial HMTs catalyze the methylation of inorganic iodide to methyl iodide, using Sadenosyl- L-methionine (SAM) as the methyl donor. The reaction involves the halide methyltransferase (HMT) enzyme facilitating a nucleophilic attack of iodide on the electrophilic methyl group of SAM, resulting in the formation of methyl iodide and S-adenosyl-L-homocysteine (SAH). This process is a key part of the natural cycle of methyl halides, with the enzyme's activity found in various bacteria, including species like *Variovorax* sp. and *Photobacterium leiognathi* [23-28].

**Microbial methylation.** Microbial methylation is a key transformation process in the iodine cycle, particularly relevant for its influence on environmental mobility. A wide variety of terrestrial and marine bacteria, including *Roseovarius* sp., *Alteromonas macleodii*, *Synechococcus* sp., *Erythrobacter* sp., *Variovorax* sp., and *Pseudomonas* sp., possess the capability to methylate iodide (I^-^) to form volatile methyl iodide (CH_3_I), as shown in Table 2 [23-26]. This biological process usually requires S-adenosyl-L-methionine (SAM) as the methyl donor [6, 27]. Methyl iodide plays a significant role as an effective carrier, facilitating the transfer of iodine from the biosphere into the atmosphere [23]. A SAM-dependent halide methyltransferase (HMT) is an enzyme that uses S-adenosylmethionine (SAM) to transfer a methyl group to various halide ions, producing a halomethane, as shown in Fig. 4 [27, 28]. Bacterial HMTs play a role in the biosynthesis of methyl halides, such as methyl iodide, from iodide, which may have roles in cellular processes. However, their exact function remains to be investigated [27, 28]. The production of methyl iodide carries dual environmental implications, impacting both atmospheric chemistry (localized ozone destruction, cloud condensation nuclei formation) and the critical issue of radionuclide migration (mobilization of I-129), because once methylated, I-129 can volatilize, spread far from its source of contamination, and subsequently accumulate in the human thyroid gland upon deposition [6].

**Accumulation and sorption.**

In contrast to methylation, microbial accumulation and sorption processes can lead to the retention and immobilization of iodine in environmental matrices. Bacteria can accumulate iodide (I^-^), a process often initiated by the oxidation of I^-^ to HIO via extracellular vanadium iodoperoxidases, followed by the transport of HIO into the bacterial cells [10, 12, 29].

Microorganisms significantly influence iodine mobility in soil systems by promoting the iodination, or covalent binding, of soil organic matter [10]. This reaction, mediated by extracellular enzymes such as peroxidases, appears to involve continuous iodination and simultaneous deiodination in aerobic systems [29, 30]. Iodide uptake by forest soils is principally related to the activity of extracellular oxidases [10]. Experimental studies using enzyme inhibitors, such as sodium azide, or antibacterial agents, like bronopol, have demonstrated a significant decrease in I-125 tracer binding in soils compared to untreated controls, providing direct confirmation of the microbial role in soil iodide uptake and immobilization [10, 31].

### Atmospheric Chemistry of Iodine

Marine iodide (I^-^) plays a crucial role in controlling atmospheric ozone levels [6]. The rapid reaction of seasurface iodide with ozone (O_3_) is believed to be the single largest source of gaseous iodine to the atmosphere [32]. Inorganic iodine emissions, particularly molecular iodine (I_2_), can be formed from iodide through various mechanisms, including the reaction: 2I^-^(aq) + O_3_ + 2H+ → H_2_O + O_2_ + I_2_(g), the Dushman reaction, and direct photooxidation of I^-^(aq) [6].

Once emitted, I_2_ gas can be photolyzed by both UV and visible wavelengths (I_2_ + hν → I + I), producing atomic iodine [6]. This photochemically produced atomic iodine then scavenges ozone (I + O_3_ → IO + O_2_), and the resulting IO radical can be photolyzed back into atomic iodine (IO + hν → I + O) or react with other species (*e.g.*, IO + O → I + O_2_; IO + NO → I + NO_2_), forming a catalytic cycle for ozone destruction [6]. Reactive iodine is a significant contributor to atmospheric ozone loss, accounting for approximately 15% of tropospheric ozone loss and up to 4.2% of stratospheric column ozone loss over the Antarctic "ozone hole" [6].

An essential consideration in this context is the potential for a climate-iodine feedback loop. It has been suggested that future climate-induced oceanographic changes could result in a significant alteration in aqueous iodide concentrations in the surface ocean, with profound implications for atmospheric air quality and climate [32]. This implies a complex feedback mechanism: climate change alters oceanographic conditions, which in turn modify iodine speciation and concentration in surface waters. This change then affects the flux of iodine to the atmosphere, impacting atmospheric ozone levels, which can, in turn, further influence radiative forcing and climate. This highlights a dynamic, long-term environmental feedback mechanism that can either exacerbate or mitigate the effects of climate change. The iodine cycle is intrinsically linked to global climate regulation through its impact on atmospheric ozone. Anthropogenic activities and climate change that alter ocean chemistry or temperature can inadvertently perturb this natural cycle, leading to unforeseen consequences for air quality and climate. This highlights the importance of climate-aware environmental management and integrated Earth system modeling in predicting future ecological states.

### Interplay of Biotic and Abiotic Processes in Iodine Cycling

The biogeochemical cycling of iodine is a complex system driven by both biotic (biological) and abiotic (physical and chemical) processes occurring across the lithosphere, hydrosphere, and atmosphere [2]. This suggests that the cycle is not simply the sum of independent biotic and abiotic components, but rather a complex system of intricate feedback loops.

For instance, hypoiodous acid (HIO), an intermediate produced by microbial activity, can undergo abiotic disproportionation into iodide (I^-^) and iodate (IO_3_^-^) [10]. Furthermore, while the abiotic oxidation of I^-^ to I_2_ or HIO is inherently very slow due to iodide's stability under typical environmental conditions, multiple studies have demonstrated that microorganisms and/or their enzymes significantly improve the kinetics of this reaction [10]. This suggests that microbial processes can directly influence and accelerate abiotic chemical transformations. On a larger, geological timescale, the marine iodine cycle has fundamentally shaped the stability and abundance of the Earth's ozone layer for approximately two billion years, illustrating a long-term co-evolution and interaction between biological and geological processes [6]. These dynamic interactions mean that changes in one component, such as microbial community composition or general environmental conditions, can have cascading effects on the entire system, which can be highly context-dependent.

**Fig. 5:**
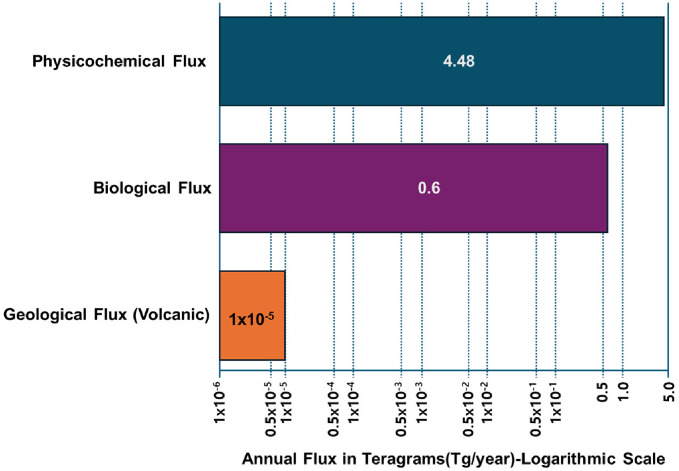
Contribution of physicochemical, biological, and geological processes in the global iodine cycle.

An integrated analysis of the iodine cycle reveals that the primary mechanism for global cycling is the highly efficient ocean-atmosphere-terrestrial linkage. Available data, derived from a synthesis of modern global models and observational studies, clearly demonstrate the dominance of ocean-based fluxes. While the data provided do not provide a single, comprehensive flux budget, comparative analysis of individual flux estimates allows for the determination of the relative magnitude of each flux contribution [11]. Fig. 5 shows the contribution of physicochemical, biological, and geological processes in the global iodine cycle. Pound et. al., presented that physicochemical flux plays a dominant role in the iodine cycle on Earth from their analyses using a coupled surface microlayer box model, and estimated that the global total for inorganic iodine emissions, primarily from the ozone-iodide reaction, is approximately 4.48 teragrams (Tg) per year [33]. They also report that biological flux plays a significant role in the iodine cycle on Earth, and an additional 0.6 Tg per year of iodine is attributed to the emission of iodinated hydrocarbons produced by marine biota, more than seven times smaller than the inorganic flux [33]. Geological flux seems to play a minor role in the iodine cycle on Earth, because Episodic volcanic eruptions, while locally and temporarily significant, contribute a negligible amount to the global annual budget. For example, a single major eruption released an estimated 10 megagrams (Mg) of iodine monoxide. This is orders of magnitude smaller than the annual marine fluxes (10 Mg vs. 4,480,000 Mg or 4.48 Tg) [34].

## Characteristics and Environmental Impact of Radioactive Iodine-129

### Origin and Persistence of Iodine-129

Iodine-129 (I-129, ^129^I) is a long-lived beta-emitting radioisotope that originates from both natural processes and, predominantly, from anthropogenic nuclear activities [35]. Table 3 summarizes some crucial properties and environmental impact of Iodine-129. The primary anthropogenic sources include releases from spent nuclear fuel reprocessing plants [36], nuclear weapons manufacturing, and effluents from nuclear power plants [8]. The impact of human nuclear activities on the global iodine isotopic balance has been substantial, with the I-129/I-127 ratios in the environment increasing dramatically from approximately 10^-12^ in the pre-nuclear era to values ranging from 10^-10^ to 10^-4^ today [35].

I-129 possesses an exceptionally long half-life of 15.7 million years, and this extreme longevity positions it as one of the most significant long-term hazards associated with nuclear waste disposal [36, 37]. The long half-life of I-129 is a unique and dominant characteristic that fundamentally redefines the difficulty of managing I-129. Because this period is far beyond human planning and institutional stability, traditional “clean-up” or “collapse to safe levels” strategies applied to many other contaminants are not feasible in the realistic human timeframes of I-129. Instead, the paradigm shifts to “long-term management” or “permanent containment and isolation.” This emphasizes the fundamental differences in managing long-lived radionuclides compared to other contaminants, underscoring the need for a shift in regulatory and societal perspectives on intergenerational responsibility.

### Environmental Mobility and Bioaccumulation of Iodine-129

I-129 exhibits high mobility within the environment, particularly in groundwater systems [35]. This high mobility is a primary concern, as it facilitates the widespread dispersion of the radioisotope from contamination sources, potentially over vast distances [6]. Its mobility is critically influenced by its chemical speciation; iodide (I^-^) is notably more mobile than iodate (IO_3_^-^) and often represents the predominant iodine species in terrestrial environments due to prevailing pH and Eh (redox) conditions [13, 38].

A significant characteristic of I-129 is its high bioaccumulation factor, with approximately 90% of all body iodine concentrating specifically in the thyroid gland, which is an essential organ for life in all vertebrates [9, 39]. The thyroid gland uses iodine to synthesize thyroid hormones (THs) that regulate a multitude of physiological processes, including metabolism, growth, and development [40]. However, the thyroid gland cannot chemically distinguish between stable iodine I-127 and its radioactive counterparts like I-129 and I-131. Consequently, any radioactive iodine that enters the body will be selectively and efficiently absorbed by the thyroid, potentially increasing the risk of thyroid cancer or other thyroid-related health issues in all vertebrates, even including fish [39].

For example, thyroid diseases, such as goiters, are prevalent in some elasmobranchs, such as sharks and rays [41]. In addition, the high-volume consumption of food products like contaminated dairy milk can lead to significant internal exposure in people and animals [39].

Data show that iodine concentrations in marine algae can range from 10 to 6,000 μg/g dry weight, with brown algae, such as members of the Fucales and Laminariales orders, demonstrating the highest values [35]. This is significantly higher than the iodine content found in freshwater algae (~0.00001% by weight) and terrestrial plants (<1 μg/g) [42]. However, the concentration ratio (CR) of iodine is much higher in freshwater fish (CR of 85-544, 0.003-0.81 ppm) than in marine fish (CR of 10-20; 0.023-0.11 ppm), indicating greater susceptibility in isolated ecosystems [42, 43]. The bioaccumulation of I-129 occurs at the base of the food web and is then transferred to higher trophic levels [42]. This is a critical observation that differentiates the hazard profile of I-129 from other radionuclides that may pose a greater threat to food safety. The accumulation of I-129 in fish is a process of environmental concern, but does not appear to represent a significant immediate public health risk from ingestion [44].

The combination of high mobility and high bioaccumulation creates a significant amplification of risk. Mobile pollutants have the potential to spread over large areas, exposing larger populations, and at the same time, due to their specific bioaccumulation properties, even low environmental concentrations can be converted into significant internal doses in sensitive organs. This explains why I-129 is designated a "primary risk driver" [37] despite its relatively low radioactivity compared to other radionuclides [9].

Despite the identification of various attenuation mechanisms, I-129 plumes, such as those at the U.S. Department of Energy Hanford Site, are projected to persist for over 150 years [45]. Current treatment technologies frequently struggle to achieve the stringent federal drinking water standards for I-129, which are exceptionally low [37]. The "dilute and disperse" approach, sometimes used for I-129, is intended to reduce concentration but inherently relies on this mobility, and still poses long-term risks due to its bioaccumulation potential [46]. The unique combination of extreme longevity, high environmental mobility, and specific bioaccumulation in the thyroid makes I-129 an exceptionally challenging radionuclide to manage. Effective remediation strategies must therefore prioritize both containment (to limit spread) and reduction of bioavailability (to limit uptake), requiring a multi-faceted and integrated approach.

### Health Effects and Risk Assessment of Iodine-129

Exposure to radioactive iodine, including I-129, can lead to various thyroid problems, such as the formation of nodules and thyroid cancer [39]. This direct health impact is primarily attributable to the thyroid gland's highly selective uptake and concentration of iodine, which effectively concentrates the radioisotope within this sensitive organ [39, 47].

I-129 is considered a primary risk driver at major radiological waste sites, such as the Hanford Site, due to its combination of an extremely long half-life, inherent toxicity, specific accumulation in the thyroid, substantial inventory at source terms, and high environmental mobility [40]. The significant hazard posed by I-129 is reflected in its very low drinking water standard (1 pCi/L) set by the U.S. Federal Registry, which is the weakest among all radionuclides [9]. A particularly striking illustration of I-129's risk amplification is provided by data from the Savannah River Site (SRS), where I-129 accounts for only 0.00002% of the radiation released offsite but contributes 13% of the population dose [9]. This represents a six orders of magnitude increase in risk due to its radioactivity.

This observation reveals that the hazard of I-129 is not simply a linear function of its radioactivity. Instead, its risk is disproportionately amplified by its unique biogeochemical properties, specifically, its high environmental mobility and its highly selective bioaccumulation in the human thyroid gland. This fundamental characteristic explains why it is assigned the lowest drinking water standard among all radionuclides, despite its relatively low specific activity. Therefore, the risk assessment and management of radionuclides, such as I-129, must extend beyond simple measurements of radioactivity. A comprehensive approach requires a deep understanding of the unique biogeochemical behavior of these radionuclides, which can significantly amplify their environmental and health impacts, and thus require multifaceted and interdisciplinary strategies for effective remediation and longterm management.

### Global Distribution of Radioactive Iodine, I-129

**Fig. 6:**
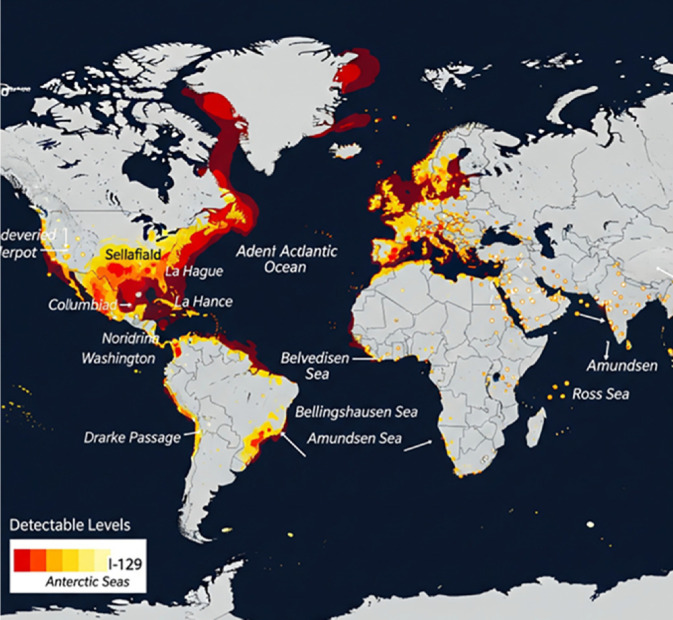
Global distribution of radioactive iodine, I-129.

The global distribution of I-129 is far from uniform, exhibiting a pronounced asymmetry [48]. Over 99% of the current mobile I-129 reservoir is concentrated in the Northern Hemisphere, directly reflecting the geographical concentration of primary anthropogenic sources, as shown in Fig. 6 [48-51]. Within this hemisphere, distinct hotspots are observed, particularly in the North Atlantic and Arctic Oceans, where powerful currents, such as the North Atlantic Current and the Norwegian Coastal Current, efficiently transport discharges from European reprocessing plants [48-51]. While less pronounced, the detection of anthropogenic I-129 in the Southern Hemisphere, including the Antarctic seas, confirms inter-hemispheric transport via global thermohaline circulation [48-51].

North Atlantic and Arctic Oceans regions are prominent hotspots for I-129 due to direct liquid discharges from the Sellafield (Irish Sea) and La Hague (English Channel) reprocessing facilities [52]. I-129 concentrations in the Nordic Seas are typically on the order of 10^9^ atoms·kg^-1^, which is one to two orders of magnitude higher than concentrations found in less affected areas of the North Atlantic Ocean [52]. The primary transport mechanism for this I-129 is the North Atlantic Current and the Norwegian Coastal Current, which carry the discharges northward [52]. Inventory estimations indicate that the North Sea holds approximately 147 kg of I-129, while the English Channel contains about 78 kg, with over 90% of this residing in the Southern Bight and the eastern English Channel [52, 53].

Coastal Pacific Ocean waters off California and the US Pacific Northwest sites show detectable levels of I-129 [49]. Notably, highly elevated I-129/I-127 values are found in the Columbia River, downstream from the decommissioned Hanford nuclear facility. Although this anthropogenic I-129 becomes significantly diluted once it reaches the broader Pacific Ocean, the signal persists in surface seawater off the west coast of the U.S., resulting in higher I-129/I-127 levels than other Pacific sites [49]. Despite extensive monitoring over the nine years since the Fukushima Daiichi accident, no I-129 signal linked to Fukushima has been positively identified in these U.S. coastal waters, suggesting significant dilution or complex, diffuse transport patterns [49].

In Antarctic Seas, anthropogenic I-129 has been detected in surface seawater samples from the Drake Passage, Bellingshausen, Amundsen, and Ross Seas in [51]. Concentrations in the Bellingshausen Sea show a decrease from greater than 2.6 × 10^6^ atoms/L at certain locations to 1.5 × 10^6^ atoms/L along specific surface sea current pathways [51].

## Microbial Bioremediation for Iodine-129

Microbial bioremediation offers several distinct advantages over conventional physicochemical methods for managing radioactive contaminants, including iodine-129. It is generally considered more environmentally friendly, cost-effective, and sustainable [8]. Unlike methods that collect and store pollutants, bioremediation is a microbiologically organized procedural activity that aims to break down or transform contaminants into less toxic or non-toxic elemental and compound forms [54, 55]. Although harmful ionizing radiation is emitted from the radioactive atomic elements themselves and cannot be eliminated by transformation into other molecules, microorganisms can effectively neutralize the toxic effects of radioactive waste by changing its chemical composition, solubility, and mobility [8]. This can involve sequestering radioactive elements through biosorption and biomineralization processes, or direct and/or indirect redox transformations [8].

**Fig. 7:**
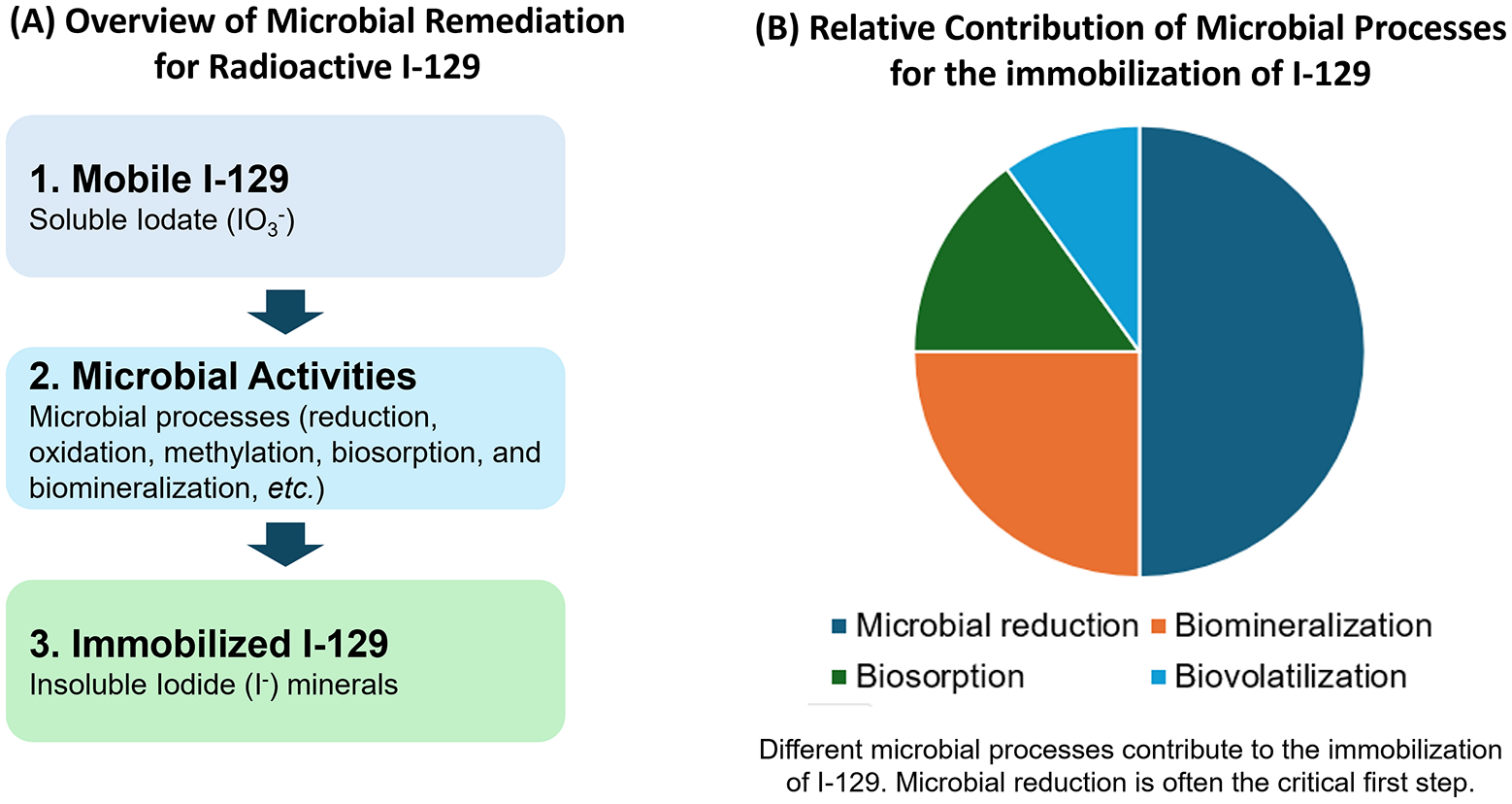
Overview of microbial bioremediation of radioactive I-129.

Bioremediation offers a sustainable strategy by utilizing naturally occurring microorganisms to transform mobile, soluble I-129 into stable, insoluble forms, effectively immobilizing it and reducing its bioavailability. The process is not a single action but a combination of powerful microbial mechanisms that work together to trap radioactive iodine, as shown in Fig. 7. Microbial bioremediation of radioactive I-129 involves using microorganisms to sequester, transform, or accumulate I-129 in the environment, mitigating its spread and toxicity. Key approaches include bioreduction, biomineralization, biosorption, and Biovolatilization by specific microbes [8]. The behavior of I-129 in the environment is complex, involving volatilization, accumulation in organisms, and redox changes, and microbial bioremediation is sensitive to environmental factors such as pH and temperature, requiring careful monitoring for optimal effectiveness [8, 13, 38]. Different microbial processes contribute to the overall immobilization of I-129. Microbial reduction is often the critical first step in this process. Particular groups of bacteria are essential for practical bioremediation [10, 22, 49]. Iodate-reducing bacteria directly convert highly soluble iodate to less soluble iodide, the crucial first step. Sulfate and iron-reducing bacteria create conditions and produce minerals (like iron sulfides) that help trap iodide through co-precipitation (biomineralization). General microbial biomass cell surfaces can absorb iodine (biosorption), while some microbes can take it up internally (bioaccumulation). Bacterial HMT (halide methyltransferase) involves the transformation of iodine into volatile organic compounds such as CH_3_I, which can then be released into the atmosphere (biovolatilization). Understanding these processes is crucial for developing effective bioremediation strategies. Research is exploring the iodine-associating abilities of both aerobic and anaerobic microorganisms to find strains that can contribute to iodine cycle management. Microbial approaches offer a sustainable way to manage I-129 in the long term by sequestering it in the environment, and nanomaterials and adsorption techniques can be combined with microbial methods to enhance iodine capture and recovery in highly contaminated sites.

Microorganisms play a crucial role in the biogeochemical cycling of iodine, influencing its speciation, solubility, and mobility [10]. Bioremediation leverages these natural microbial processes to transform or immobilize radionuclides. The key mechanisms involved in microbial iodine bioremediation include:

### Microbial Reduction

Microbial reduction is a key mechanism that impacts iodine speciation and mobility. Dissimilatory IO_3_^-^- reducing bacteria (DIRB) are capable of reducing iodate (IO_3_^-^) to iodide (I^-^) directly under anoxic conditions [10]. This process involves specific iodate reductases, such as periplasmic iodate reductase IdrABP1P2, extracellular DMSO reductase DmsEFAB, and metal reductase MtrCAB [10]. These enzymes initially reduce IO_3_^-^ to hypoiodous acid (HIO) and hydrogen peroxide (H_2_O_2_); HIO is then proposed to disproportionate abiotically into I^-^ and IO_3_^-^ at a 2:1 ratio [10]. The H_2_O^2^ produced is subsequently reduced to H_2_O by IdrP1P2 and MtrCAB as a detoxification mechanism [10]. Additionally, dissimilatory Fe(III)- and sulfate-reducing bacteria contribute to IO_3_^-^ reduction, either directly via their own IO_3_^-^ reductases or indirectly via the abiotic reduction by their metabolic byproducts, Fe(II) and sulfide [10]. Examples of bacteria demonstrating this capability include *Pseudomonas* sp. SCT, *Shewanella oneidensis*, *Desulfovibrio desulfuricans*, and *Shewanella putrefaciens*, which have been shown to reduce iodate under anaerobic conditions [14-17,19]. The reduction of iodate to iodide can increase the mobility of iodine; however, iodide is generally more mobile than iodate [16]. Therefore, understanding and controlling this conversion is crucial. It represents a key step in the iodine cycle that can be manipulated to influence the overall fate and transport of I-129 in contaminated systems.

### Biosorption and Bioaccumulation

Microbes can absorb iodine onto their cell surfaces (biosorption) or take it up into their cells (bioaccumulation). This process can effectively remove iodine from the aqueous phase and immobilize it. The cell walls of bacteria, with their various functional groups, can act as binding sites for metal ions, including iodine [56]. Bacteria accumulate iodide (I^-^), which is oxidized to HIO by their extracellular vanadium iodoperoxidases, and the HIO is then transported into the bacterial cells [10]. Studies on iodide-accumulating bacteria from marine sediments and contaminated aquifers, such as *Flexibacter aggregans*, *Arenibacter troitsensis*, *Streptomyces/Kitasatospora* spp., *Bacillus mycoides*, and *Ralstonia/Cupriavidus* spp., have demonstrated their capacity to concentrate iodide, albeit at varying efficiencies depending on the environment [13].

### Biomineralization (Bioprecipitation)

This process involves the precipitation of radionuclides through microbial ligands, forming stable biogenic minerals that retain radioactive contaminants [8]. For I-129 specifically, microbial promotion of iodination, which is the covalent binding of iodine to soil organic matter, is a key immobilization mechanism [10]. This reaction is mediated by extracellular enzymes, such as peroxidases, provided by microbial activity [57]. Experimental evidence from studies using enzyme inhibitors or antibacterial agents has shown a significant decrease in I-125 tracer binding in soils, directly confirming the microbial role in iodide uptake and immobilization in soils [10]. These immobilization mechanisms are particularly advantageous for I-129 given its exceptionally long half-life, as they offer a means to reduce its mobility and bioavailability over geological timescales.

### Biovolatilization

Microbial biovolatilization involves the transformation of iodine into volatile organic compounds, which can then be released into the atmosphere. The primary mechanism for this is the microbial methylation of iodide (I^-^) to form methyl iodide (CH_3_I) [2]. A wide variety of terrestrial and marine bacteria are capable of this process, often utilizing S-adenosyl-L-methionine as the methyl donor [6]. Methyl iodide plays a significant role as an effective carrier, facilitating the transfer of iodine from the biosphere into the atmosphere [2].

For I-129, biovolatilization presents a dual nature. On the one hand, it could be considered a mechanism for removing radionuclides from localized contaminated sources, especially in aquatic or soil environments. However, the subsequent atmospheric transport of volatile I-129, once methylated, allows it to spread far from the contaminated area. Upon deposition, this volatile I-129 can then accumulate in the human thyroid gland, posing a health risk [6]. This means that while it removes the contaminant from one compartment, it disperses it to another, potentially increasing the overall exposure risk over wider areas. Studies at the Hanford Site have shown that iodide volatilization activity was consistently higher under native, oligotrophic (nutrient-poor) sediment conditions, and carbon and nutrient supplementation led to a significant reduction in methyl-iodide formation [10]. This observation is critical for remediation strategies, as it suggests that simple biostimulation aimed at enhancing overall microbial activity may inadvertently suppress methylation, which could be beneficial if the goal is to prevent atmospheric dispersal; however, it also complicates strategies if removal from the source is the primary objective.

### Efficacy of Bioremediation

Bioremediation, including microbial bioremediation and phytoremediation, is an emerging field that offers a potentially more ecological and economic alternative to traditional conventional methods. A comparison of the quantitative performance reveals a clear difference in the maturity and application of conventional and bioremediation methods, as shown in Table 4. Conventional methods, particularly for gas-phase streams, have a robust body of data demonstrating high, repeatable efficiencies. For high-concentration, controlled waste streams, such as nuclear fuel reprocessing, conventional methods such as caustic scrubbing or silver-based adsorbents provide a high level of quantitative certainty, with removal factors and efficiencies often exceeding 99% [58]. However, this performance comes with significant drawbacks: high costs and the generation of hazardous secondary waste [8, 66]. Conventional methods, while effective in controlled environments, struggle to scale to the sheer size and complexity of large, dilute plumes. The cost of materials and the logistics of pump-and-treat systems for vast contaminated areas are often prohibitive [45]. Bioremediation, by contrast, is a cost-effective and environmentally benign alternative that theoretically addresses the speciation challenge by converting mobile iodate to less mobile iodide [62]. Microbial bioremediation and phytoremediation, as in-situ processes, are conceptually well-suited for such large, diffuse plumes and offer a significant cost advantage [64, 65]. However, the lack of data on their longevity and predictability in complex subsurface environments raises substantial concerns about their real-world implementability and long-term effectiveness.

## Challenges and Future Directions of Microbial Bioremediation for Iodine-129

Despite its potential, microbial bioremediation of I-129 faces several challenges: 1) Long half-life of I-129: The extremely long half-life of I-129 means that any remediation strategy needs to be effective over geological timescales, which is a significant hurdle for any technology, including bioremediation, 2) Uncertainty in biogeochemical fate and transport: There is still uncertainty regarding the exact biogeochemical fate and transport of I-129 in the environment. A more comprehensive understanding of these processes is crucial for developing effective bioremediation strategies, 3) Low concentration and speciation: I-129 often exists in dilute plumes in groundwater, and its speciation (*e.g.*, iodate, iodide, organo-iodine) can vary, impacting the effectiveness of different microbial processes, 4) Site-specific conditions: The efficacy of bioremediation is highly dependent on site-specific conditions such as soil type, geochemistry, nutrient availability, and the presence of indigenous microbial populations, 5) Scalability and long-term Effectiveness: Translating laboratory-scale successes to large-scale field applications and ensuring long-term effectiveness are significant challenges for bioremediation technologies, and 6) Monitoring and verification: Monitoring the long-term impact of bioremediation on I-129 concentrations and ensuring compliance with stringent drinking water standards (*e.g.*, 1 pCi/L) can be difficult.

**Fig. 8:**
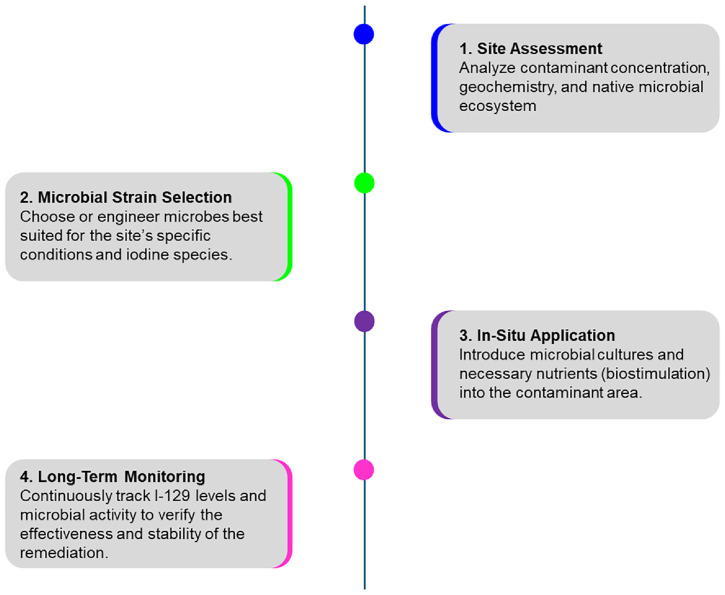
Radioactive I-129 microbial bioremediation strategy for long-term stewardship to manage and mitigate risk over geological time scales.

Implementing a microbial bioremediation strategy requires a systematic, multi-stage approach, from initial site evaluation to long-term monitoring to ensure the contaminant remains safely immobilized, as displayed in Fig. 8. Firstly, it requires analyzing contaminant concentration, geochemistry, and the native microbial ecosystem of the contaminant area for site assessment. Secondly, it requires a highly performing microbial strain, which can be achieved by selecting effective microbes or genetically engineering the microbes best suited for the site’s specific conditions and iodine species. The use of genetically engineered microbial bioremediation has been explored to address the I-129 challenge [27, 68]. Rather than relying solely on physical barriers, this approach seeks to leverage and enhance natural microbial pathways to transform the radioactive I-129 into an immobilized form at the molecular level. A synthesis of research from radiochemistry, environmental engineering, and molecular biology reveals that specific molecular mechanisms, such as dissimilatory iodate reduction and biovolatilization, offer a targeted and practical pathway for remediation [8, 67]. The report highlights the potential of a strategic fusion: introducing the key gene for this pathway, IO_3_^−^ reductase (*idrA*), into a robust, radiation-resistant microbial host like *Deinococcus radiodurans* [67]. Bayer *et al*., chemically synthesized all 89 putative halide methyltransferase (HMT) genes from plants, fungi, bacteria, and unidentified organisms present in the NCBI sequence database using a synthetic metagenomic approach, demonstrating high production of methyl halides by engineered microbes [27]. The biologically engineered system could be further enhanced by synergistic technologies, such as the use of biogenic gold nanoparticles, which exhibit a strong chemical affinity for iodine and can act as an irreversible sink [68]. Next, it requires *in-situ* application by introducing microbial cultures and necessary nutrients (biostimulation) into the contaminant area and long-term monitoring by continuous tracking of I-129 levels and microbial activity to verify the effectiveness and stability of the remediation. Sometimes, a hybrid approach, combining the high efficiency of conventional methods, may offer the most promising path forward, especially for contained streams with emerging, in-situ alternatives for diffuse plumes.

Concurrently, fundamental research is required to address the knowledge gaps in bioremediation, specifically regarding its long-term viability and the factors that govern the fate and transport of iodine in natural systems [69]. The "low practicability" conclusion for all candidate technologies at sites like Hanford [69] suggests a need to re-evaluate what constitutes a "remedial success" for long-lived, highly mobile radionuclides. Microbial bioremediation of radioactive iodine-129 could be key player to shift from complete plume removal to long-term stewardship strategies that manage and mitigate risk over geological time scales.

## Figures and Tables

**Table 1:** Iodine Speciation and Distribution Across Environmental Compartments.

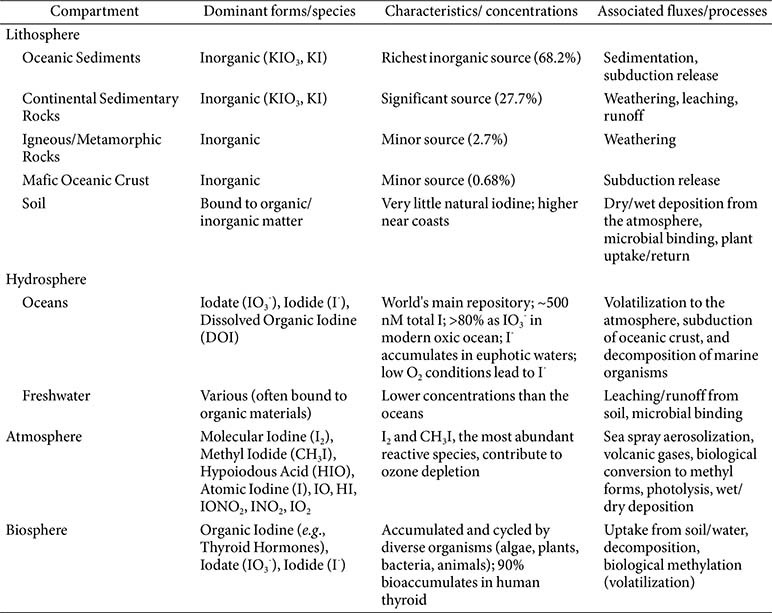

**Table 2:** Bacterial species and their characteristics known to produce methyl iodide.

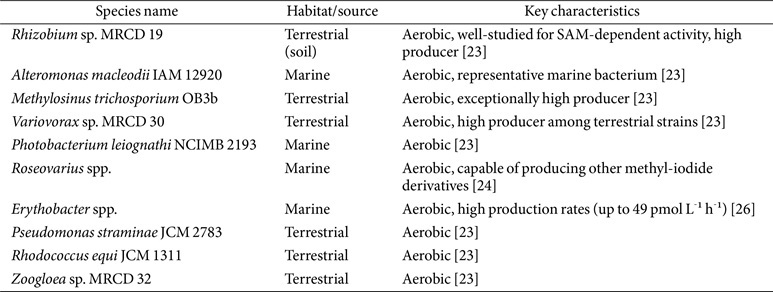

**Table 3:** Characteristics and Environmental Impact of Iodine-129.

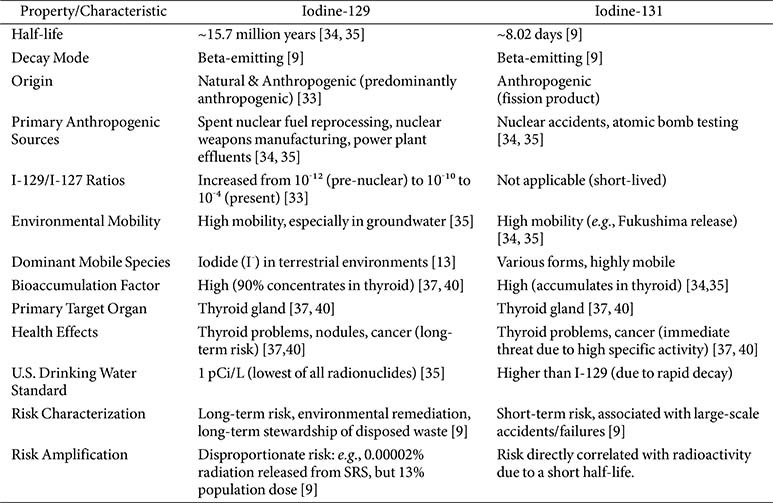

**Table 4:** Comparison of the quantitative performance of conventional and bioremediation methods for radioactive iodine-129.

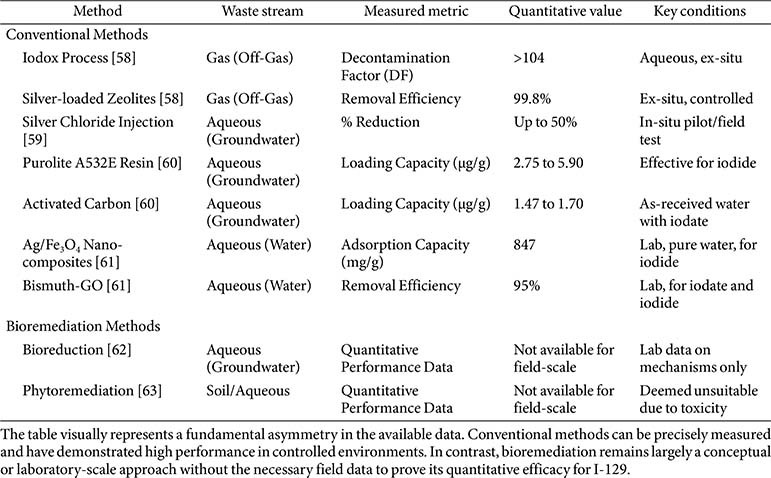

## References

[1] Elderfield H, Truesdale VW (1980). On the biophilic nature of iodine in seawater. Earth Planet. Sci. Lett..

[2] Espino-Vázquez AN, Rojas-Castro FC, Fajardo-Yamamoto LM (2022). Implications and practical applications of the chemical speciation of iodine in the biological context. Future Pharm..

[3] Whitehead, DC. 1984. The distribution and transformations of iodine in the environment. *Environ. Int.* **10:** 321-339. 10.1016/0160-4120(84)90139-9

[4] Moreda-Pineiro A, Romaris-Hortas V, Bermejo-Barrera P (2011). A review on iodine speciation for environmental, biological, and nutrition fields. J. Anal. Atomic Spect..

[5] Ahad F, Ganie SA (2010). Iodine, iodine metabolism, and iodine deficiency disorders revisited. Indian J. Endocrinol. Metab..

[6] Liu J, Hardisty DS, Kasting JF, Fakhraee M, Planavsky NJ (2025). Evolution of the iodine cycle and the late stabilization of the earth's ozone layer. Proc. Natl. Aca. Sci. USA.

[7] Carpenter LJ, Chance RJ, Sherwen T, Adams TJ, Ball SM, Evans MJ (2021). Marine iodine emissions in a changing world. Proc. Math. Phys. Eng. Sci..

[8] Calvillo JM. 2025. How Do Microbes Remove Radioactive Waste? pp.27-32. *In Microcosm*. Spring 2025. American Society of Microbiology, Washington, D.C.

[9] Kaplan DI, Denham ME, Zhang S, Yeager C, Xu C, Schwehr (2014). Radioiodine biogeochemistry and prevalence in groundwater. Crit. Rev. Environ. Sci. Technol..

[10] Yeager CM, Amachi S, Grandbois R, Kaplan DI, Xu C, Schwehr KA (2017). Microbial transformation of iodine: from radioisotopes to iodine deficiency. Adv. Appl. Microbiol..

[11] Wikipedia. 2025. Iodine cycle. Available from https://en.wikipedia.org/wiki/Iodine_cycle. Accessed July 20, 2025.

[12] Amachi S (2008). Microbial contribution to global iodine cycling: volatilization, accumulation, reduction, oxidation, and sorption of iodine. Microbes Environ..

[13] Li HP. 2012. Roles of Naturally Occurring Bacteria in Controlling Iodine-129 Mobility in Subsurface Soils (Doctoral dissertation).

[14] Toporek YJ, Mok JK, Shin HD, Lee BD, Lee MH, DiChristina TJ (2019). Metal reduction and protein secretion genes required for iodate reduction by *Shewanella oneidensis*. Appl. Environ. Microbiol..

[15] Shin HD, Toporek Y, Mok JK, Maekawa R, Lee BD, Howard MH, *et al*. 2022. Iodate reduction by *Shewanella oneidensis* requires genes encoding an extracellular dimethylsulfoxide reductase. *Front. Microbiol.* **13:** 852942. 10.3389/fmicb.2022.852942 35495678 PMC9048795

[16] Guo J, Jiang Y, Hu Y, Jiang Z, Dong Y, Shi L (2022). The roles of DmsEFAB and MtrCAB in extracellular reduction of iodate by *Shewanella oneidensis* MR‐1 with lactate as the sole electron donor. Environ. Microbiol..

[17] Hou L, Zheng B, Jiang Z, Hu Y, Shi L, Dong Y, *et al*. 2024. The dmsEFABGH operon encodes an essential and modular electron transfer pathway for extracellular iodate reduction by *Shewanella oneidensis* MR-1. *Microbiol. Spectrum.* **12:** e00512-24. 10.1128/spectrum.00512-24 38916364 PMC11302344

[18] Yamazaki C, Kashiwa S, Horiuchi A, Kasahara Y, Yamamura S, Amachi S (2020). A novel dimethylsulfoxide reductase family of molybdenum enzyme, Idr, is involved in iodate respiration by *Pseudomonas* sp. SCT. Environ. Microbiol..

[19] Councell TB, Landa ER, Lovley DR (1997). Microbial reduction of iodate. Water Air Soil Poll..

[20] Suzuki M, Eda Y, Ohsawa S, Kanesaki Y, Yoshikawa H, Tanaka K (2012). Iodide oxidation by a novel multicopper oxidase from the alphaproteobacterium strain Q-1. Appl. Environ. Microbiol..

[21] Shiroyama K, Kawasaki Y, Unno Y, Amachi S (2015). A putative multicopper oxidase, IoxA, is involved in iodide oxidation by *Roseovarius* sp. strain A-2. Biosci. Biotechnol. Biochem..

[22] Jiang Z, Jiang Y, Hu Y, Dong Y, Shi L (2024). The crucial and versatile roles of bacteria in global biogeochemical cycling of iodine. Geo-Bio Interfaces.

[23] Amachi, S, Kamagata, Y, Kanagawa, T, Muramatsu, Y. 2001. Bacteria mediate methylation of iodine in marine and terrestrial environments. *Appl. Environ. Microbiol.* **67:** 2718-2722. 10.1128/AEM.67.6.2718-2722.2001 11375186 PMC92930

[24] Fuse H, Inoue H, Murakami K, Takimura O, Yamaoka Y (2003). Production of free and organic iodine by *Roseovarius* spp. FEMS Microbiol. Lett..

[25] Hughes, C, Franklin, DJ, Malin, G. 2011. Iodomethane production by two important marine cyanobacteria: *Prochlorococcus marinus* (CCMP 2389) and *Synechococcus* sp.(CCMP 2370). *Mar. Chem.* **125:** 19-25. 10.1016/j.marchem.2011.01.007

[26] Fujimori T, Yoneyama Y, Taniai G, Kurihara M, Tamegai H, Hashimoto S (2012). Methyl halide production by cultures of marine proteobacteria *Erythrobacter* and *Pseudomonas* and isolated bacteria from brackish water. Limnol. Oceanogr..

[27] Bayer TS, Widmaier DM, Temme K, Mirsky EA, Santi DV, Voigt CA (2009). Synthesis of methyl halides from biomass using engineered microbes. J. Am. Chem. Soc..

[28] Tang Q, Pavlidis IV, Badenhorst CP, Bornscheuer UT (2021). From natural methylation to versatile alkylations using halide methyltransferases. ChemBioChem..

[29] Lusa M, Bomberg M, Aromaa H, Knuutinen J, Lehto J (2015). Sorption of radioiodide in an acidic, nutrient-poor boreal bog: insights into the microbial impact. J. Environ. Radioact..

[30] Behrens H (2006). Biogeochemistry of iodine in aquatic and terrestrial systems. Geophys. Res. Abs..

[31] Duborská E, Urík M, Bujdoš M, Matulová M (2019). Influence of physicochemical properties of various soil types on iodide and iodate sorption. Chemosphere..

[32] Carpenter LJ, Chance RJ, Sherwen T, Adams TJ, Ball SM, Evans MJ (2021). Marine iodine emissions in a changing world. Proc. Math. Phys. Eng. Sci..

[33] Pound RJ, Brown LV, Evans MJ, Carpenter LJ (2024). An improved estimate of inorganic iodine emissions from the ocean using a coupled surface microlayer box model. Atmos. Chem. Phys..

[34] Schönhardt A, Richter A, Theys N, Burrows JP (2017). Space-based observation of volcanic iodine monoxide, Atmos. Chem. Phys..

[35] Hou X, Hansen V, Aldahan A, Possnert G, Lind OC, Lujaniene G (2009). A review on speciation of iodine-129 in the environmental and biological samples. Anal. Chim. Acta.

[36] Kadowaki M, Katata G, Terada H, Suzuki T, Hasegawa H, Akata N (2018). Impacts of anthropogenic source from the nuclear fuel reprocessing plants on global atmospheric iodine-129 cycle: A model analysis. Atmos. Environ..

[37] Kaplan DI, Yeager C, Denham ME, Zhang S, Xu C, Schwehr KA, *et al*. 2012. *Biogeochemical Considerations Related to the Remediation of I-129 Plumes* (No. SRNL-STI-2012-00425; RPT-DVZ-AFRI-002). Savannah River Site (SRS), Aiken, SC (United States). 10.2172/1051571

[38] Gómez Martín JC, Saiz‐Lopez A, Cuevas CA, Baker AR, Fernández RP (2022). On the speciation of iodine in marine aerosol. J. Geophys. Res. Atmos..

[39] Deal CK, Volkoff H (2020). The role of the thyroid axis in fish. Front. Endocrinol (Lausanne).

[40] Wheaton CJ, Sullivan KE, Bassiouny E, Burns CM, Smukall MJ, Hendon JM (2025). Investigation of serum thyroid hormones, iodine, and cobalt concentrations across common aquarium-housed elasmobranchs. Front. Vet. Sci..

[41] Zaletel K, Mihovec A, Gaberscek S (2024). Characteristics of exposure to radioactive iodine during a nuclear incident. Radiol. Oncol..

[42] Toxicological Profile for Iodine. Atlanta (GA): Agency for Toxic Substances and Disease Registry (US); 2004 Apr. 6, POTENTIAL FOR HUMAN EXPOSURE. https://www.ncbi.nlm.nih.gov/books/NBK598103/. 38091462

[43] Teien HC, Wada T, Kashparov V, Lopez-Gutierrez JM, Garcia-Tenorio R, Hinton TG (2023). Transfer of 129I to freshwater fish species within Fukushima and Chernobyl exclusion zones. J. Environ. Rad..

[44] Sunday JS, Sowunmi AA, Akomolafe IR, Jibiri NN (2025). Evaluation of radiological risks from radionuclides in fish and sediment of Eleyele Reservoir, Ibadan, Nigeria. Environ Health Insights.

[45] Truex MJ, Freedman VL, Pearce CI, Szecsody JE. 2019. *Assessment of Technologies for I-129 Remediation in the 200-UP-1 Operable Unit* (No. PNNL-29148). Pacific Northwest National Lab.(PNNL), Richland, WA (USA). 10.2172/1593516

[46] Robshaw TJ, Turner J, Kearney S, Walkley B, Sharrad CA, Ogden MD (2021). Capture of aqueous radioiodine species by metalated adsorbents from waste streams of the nuclear power industry: a review. SN Appl. Sci..

[47] World Health Organization. 2017. Iodine thyroid blocking: Guidelines for use in planning and responding to radiological and nuclear emergencies. In *Iodine thyroid blocking: guidelines for use in planning and responding to radiological and nuclear emergencies.* 29630192

[48] Snyder G, Fehn U 2004. Global distribution of ^129^I in rivers and lakes: implications for iodine cycling in surface reservoirs. *Nucl. Instrum. Methods Phys. Res.* **223:** 579-586. 10.1016/j.nimb.2004.04.107

[49] Jull AJT, Chang CC, Burr GS, Li C, Biddulph D, Russell J, *et al*. 2021. Measurements of ^129^I in coastal Pacific Ocean waters in California and US Pacific Northwest sites. pp. 40.

[50] Maksyutov S, Patra PK, Onishi R, Saeki T, Nakazawa T (2008). NIES/FRCGC global atmospheric tracer transport model: Description, validation, and surface sources and sinks inversion. Earth Simul..

[51] Xing S, Hou X, Aldahan A, Possnert G, Shi K, Yi P, Zhou W (2017). Water circulation and marine environment in the Antarctic traced by speciation of 129I and 127I. Sci. Rep..

[52] He P, Aldahan A, Possnert G, Hou XL (2013). A summary of global 129I in marine waters. Nucl. Inst. Meth. Phys. Res..

[53] He P. 2013. *Iodine Isotopes and their Specie s in Surface Water from the North Sea to the Northeastern Atlantic Ocean* (Doctoral dissertation, Acta Universitatis Upsaliensis).

[54] Abatenh E, Gizaw B, Tsegaye Z, Wassie M (2017). The role of microorganisms in bioremediation-a review. Open J. Environ. Biol..

[55] Ayilara MS, Babalola OO (2023). Bioremediation of environmental wastes: the role of microorganisms. Front. Agron..

[56] Duborská E, Vojtková H, Matulová M, Šeda M, Matúš P (2023). Microbial involvement in iodine cycle: mechanisms and potential applications. Front. Bioeng. Biotechnol..

[57] Behrens H. 2006. Biogeochemistry of iodine in aquatic and terrestrial systems. In Geophys. Res. Abst. Vol. 8. pp. 1-1.

[58] Haefner D. 2007. Methods of Gas Phase Capture of Iodine from Fuel Reprocessing Off-Gas: A Literature Survey. https://doi.org/10.2172/911962. 10.2172/911962

[59] SRS Deploys Innovation to Clean Up Groundwater Contamination - Department of Energy, ttps://www.energy.gov/em/articles/srsdeploys-innovation-clean-groundwater-contamination.

[60] Parker, KE, Golovich, EC, Wellman, DM. 2014. Iodine adsorption on ion-exchange resins and activated carbons: batch testing. 10.2172/1163822

[61] Zia MR, Park JA, Kim JY, Kim KI, Mushtaq S (2024). A Review of Nanoparticles Utilized in the Removal of Radioactive Iodine from Wastewater Streams. J. Radiopharm. Mol. Probes.

[62] Guido-Garcia F, Law GTW, Lloyd JR, Lythgoe P, Morris K (2015). Bioreduction of iodate in sediment microcosms. Mineral. Mag..

[63] Hemming SD, Purkis JM, Warwick PE, Cundy AB (2023). Current and emerging technologies for the remediation of difficult-tomeasure radionuclides at nuclear sites. Environ. Sci. Proc. Impacts.

[64] Why Bioremediation, https://jrwbioremediation.com/whybioremediation/.

[65] Kuppan N, Padman M, Mahadeva M, Srinivasan S, Devarajan R (2024). A comprehensive review of sustainable bioremediation techniques: Eco-friendly solutions for waste and pollution management. Waste Manag. Bull..

[66] Bioremediation of radioactive waste - Wikipedia, https://en.wikipedia.org/wiki/Bioremediation_of_radioactive_waste.

[67] Reyes-Umana V, Henning Z, Lee K, Barnum TP, Coates JD (2022). Genetic and phylogenetic analysis of dissimilatory iodate-reducing bacteria identifies potential niches across the world's oceans. ISME J..

[68] Choi MH, Shim HE, Yun SJ, Park SH, Choi DS, Jang BS (2016). Gold-nanoparticle-immobilized desalting columns for highly efficient and specific removal of radioactive iodine in aqueous media. ACS Appl. Material. Inter..

[69] Santschi PH, Xu C, Zhang S, Ho Y, Li H, Schwehr K, Kaplan DI. 2012. *Laboratory report on iodine (129I and 127I) speciation, transformation and mobility in Handford groundwater, suspended particles and sediments* (No. SRNL-STI-2012-00592). Savannah River Site (SRS), Aiken, SC (United States).

